# Supplementary material for: The Intermediate Filament Synemin Regulates Non-Homologous End Joining in an ATM-Dependent Manner
Source: Cancers (Basel). 2020 Jun 28;12(7):1717. doi: 10.3390/cancers12071717 (PMC7407367; doi:10.3390/cancers12071717)
Supplement: Supplementary file 1 [file cancers-12-01717-s001.pdf]

Supplementary Materials:

The Intermediate Filament Synemin Regulates Non-Homologous End Joining in an ATM-Dependent Manner

Sara Sofia Deville, Anne Vehlow, Sarah Förster, Ellen Dickreuter, Kerstin Borgmann and Nils Cordes

Supplementary Materials and Methods

Total Protein Extraction, Western Blotting

Cells were lyzed with lysis buffer supplemented with protease inhibitor (Complete protease inhibitor cocktail from Roche, Mannheim, Germany) and phosphatase inhibitors (Na3VO4 and NaF from Sigma-Aldrich, Taufkirchen, Germany). The lysates were then incubated for 30 minutes on ice; the cell membranes where then broken using a syringe and 1 h later centrifuged at 13,000 × g for 20 min to remove debris. Since in 3D lysates it is not possible to quantify protein levels with common protein quantification kits, a gel for β-actin was necessary to evaluate the protein levels. After β-actin quantification using imageJ, proper dilutions were prepared. The chemiluminescent detection was preformed using ECL™ Prime Western Blotting System (Sigma-Aldrich).

Foci Assay

Twenty-four hours after irradiation, cells were isolated using trypsin/EDTA, fixed with 3% formaldehyde/phosphate-buffered saline (Merck, Darmstadt, Germany) and permeabilized with 0.25% Triton-X-100/phosphate-buffered saline (Roth, Karlsruhe, Germany). Staining was accomplished with specific antibodies and Vectashield/4′-6-diamidino-2-phenylindole (Alexis, Lörrach, Germany) was used as mounting medium. Foci were counted microscopically with an AxioImager A1 plus fluorescence microscope (Carl Zeiss, Jena, Germany) under a ×40 objective. Immunofluorescence images were sustained using LSM 510 meta (Carl Zeiss) or AxioImager M1 (Carl Zeiss). For testing the impact of chemotherapy and radiochemotherapy, cells were transfected with esiRNA and next day embedded into 0.5 mg/ml lrECM in 24-well plates. Twenty-four hours after 1.7 µM of Cisplatin or DMSO was added to the cells. After 1 h, cells were exposed to 6 Gy X-rays or left untreated. On the next day, cells were fixed and stained for residual foci (residual = 24 h after irradiation). For foci kinetics, cells were seeded, transfected with esiRNA on the following day and fixed at 0.5, 1, 2, 6 and 24 h after irradiation. The staining was performed as published Dickreuter et al., 2016.

Proximity Ligation Assay (PLA)

4 × 10^5 cells were irradiated with 6 Gy one day after plating. At 1 h post irradiation, cells were fixed with cold methanol for 15 min at -20°C and incubated with primary antibodies (Desmuslin, #211630 and DNA-PKcs S2056 #ab18192 from Abcam, Cambridge, UK) overnight. PLA was accomplished using ligation and amplification buffers as recommended by the manufacturer and as published [2]. Samples were analyzed with Axioimager M1 (Carl Zeiss) with a magnification of 40×.

Immunoprecipitation

4.5 × 10^6 SAS cells stably transfected with mCherry-C1 and mCherry-Synemin were harvested using cell lysis buffer (Cell Signaling, Frankfurt a. M., Germany) supplemented with 40 µL/mL Complete protease inhibitor cocktail. The total protein amount was measured by BCA assay. Cell lysates were pre-cleared using 50 µL of Protein A/G sepharose slurry (50 % v/v). To do this, the lysate-

bead solution was rotated at 4 °C for 1 h using a laboratory rotator. Following pre-clearing, lysates were centrifuged at 500 × g for 5 min and the supernatant was transferred to a new reaction tube. Primary antibodies (IgG as isotype control) were added to 1 mg protein lysate and rotated for 1 h at 4 °C. Subsequently, 50 µL of Protein A/G sepharose slurry (50% v/v) was added and rotated overnight at 4 °C. Immunoprecipitates were washed once with 600 µL of ice cold lysis buffer. Whole cell lysates and immunoprecipitated proteins were boiled in 50 µL sample buffer, separated by SDS-PAGE, transferred, and blotted. Protein precipitates were analyzed with specific primary antibodies as indicated previously.

Supplementary Figures

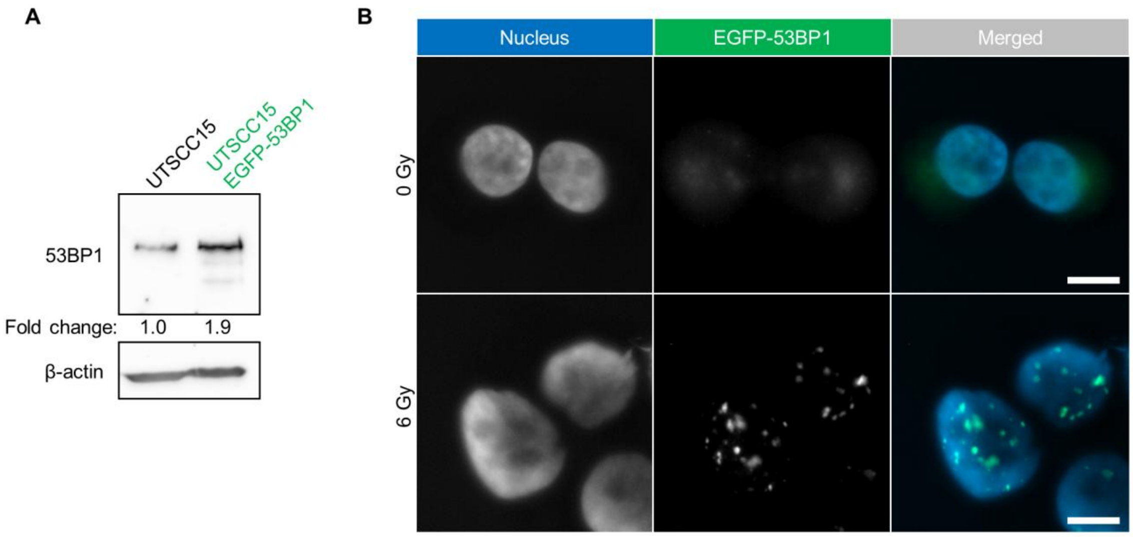

Figure S1. UTSCC15 cells stably expressing EGFP-53BP1. (**A**) Western blot of EGFP-53BP1 in whole cell lysates from UTSCC15 EGFP-53BP1 cells. Fold changes of 53BP1 expression are shown. β-actin was used as loading control. (**B)** Immunofluorescence images of cells expressing the exogenous construct after sham or 6 Gy X-ray exposure (time point = 24 h post irradiation). Nuclei were stained with DAPI.

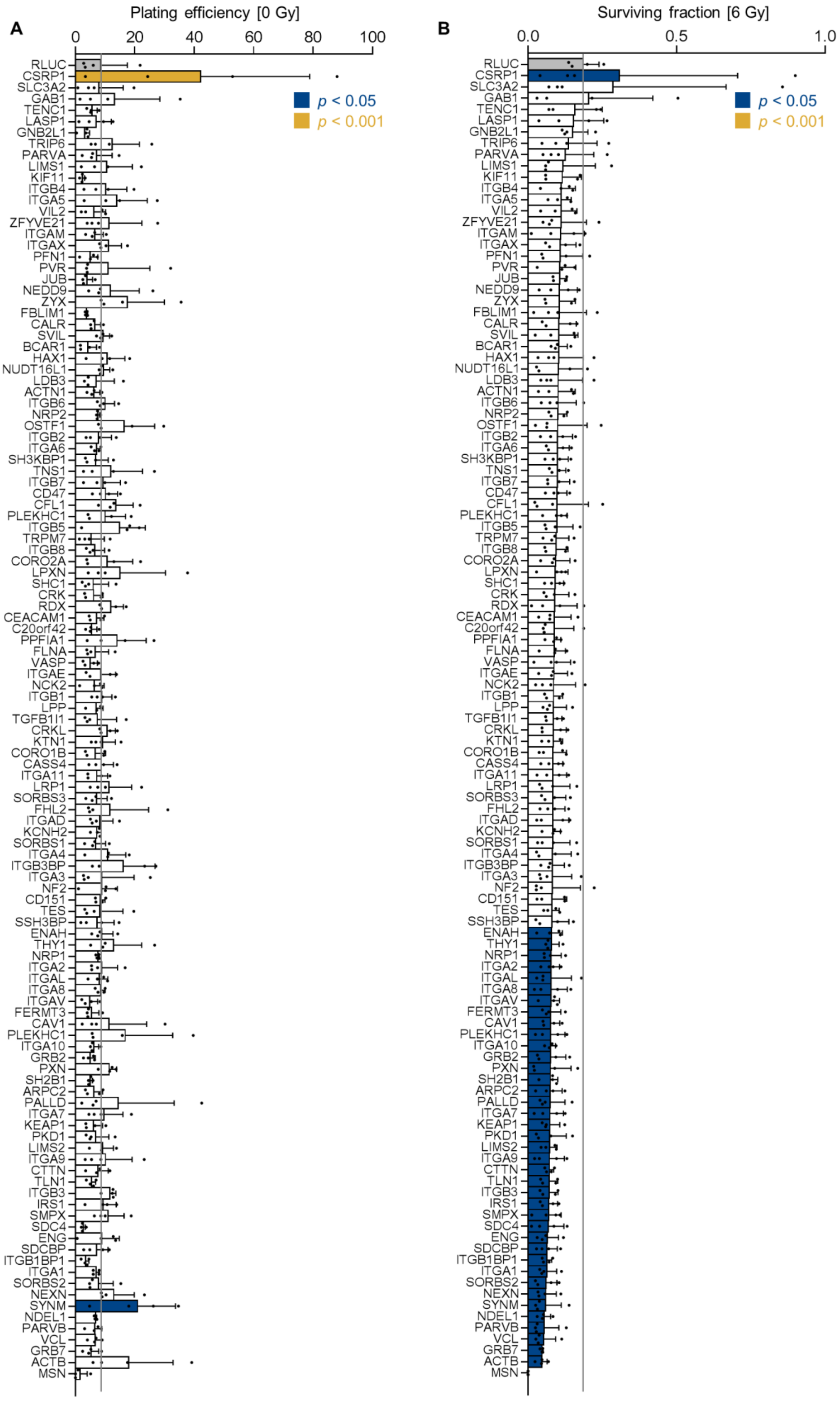

Figure S2. Identification of focal adhesion proteins (FAP) affecting cell survival and radiosensitivity. (**A**) Plating efficiency of 3D lrECM cell cultures with indicated knockdowns (n = 4). (**B)** Surviving fraction of 3D lrECM cell cultures with indicated knockdowns and 6 Gy X-rays (n = 4). Data are presented as mean ± SD.

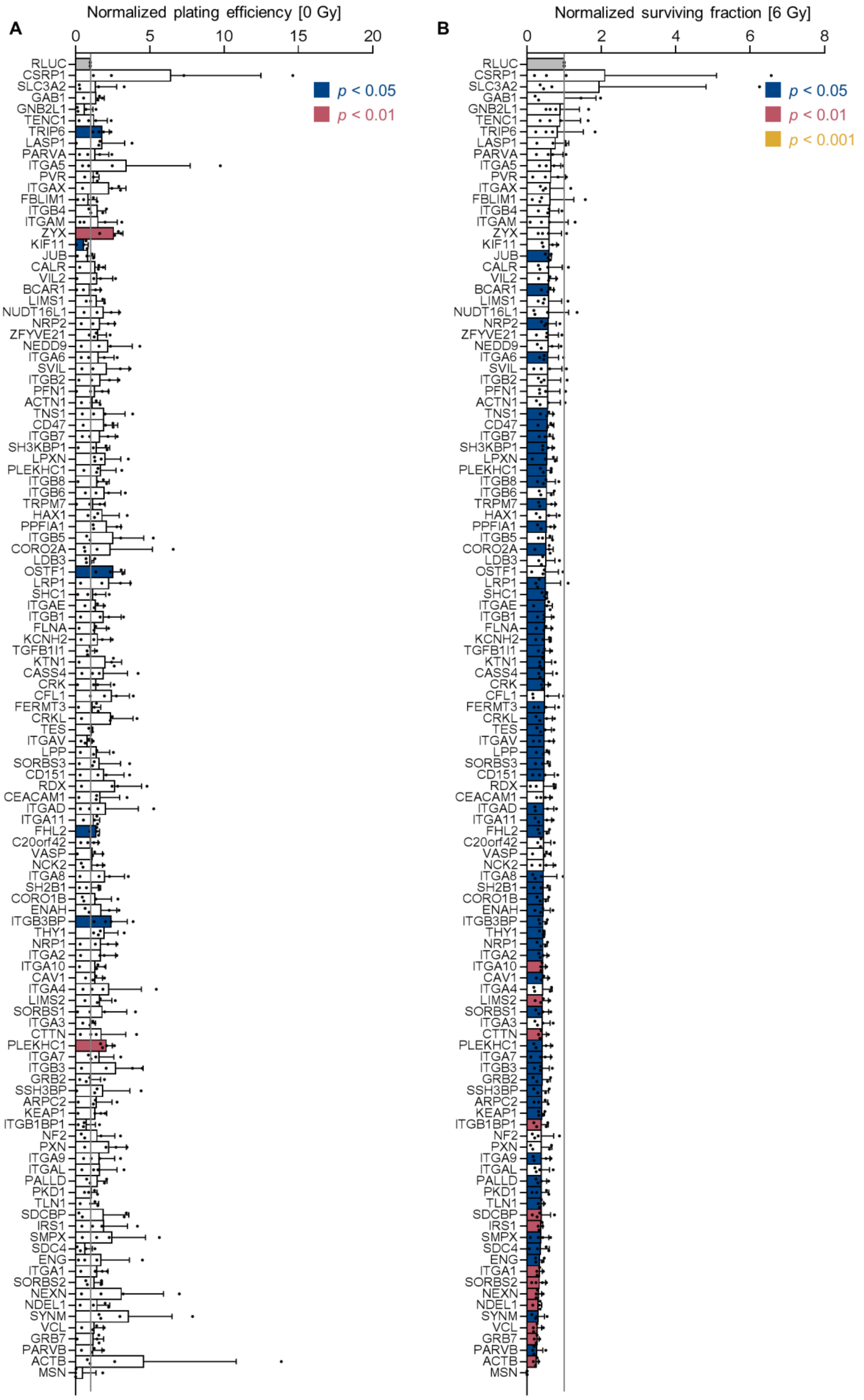

Figure S3. Identification of focal adhesion proteins (FAP) affecting cell survival and radiosensitivity. **(A)** Normalized plating efficiency of 3D lrECM cell cultures with indicated knockdowns (n = 4). (**B)** Normalized surviving fraction of 3D lrECM cell cultures with indicated knockdowns and 6 Gy X-rays (n = 4). Data are presented as mean ± SD (two-sided t-test; *$p < 0.05$, **$p < 0.01$, *** $p < 0.001$).

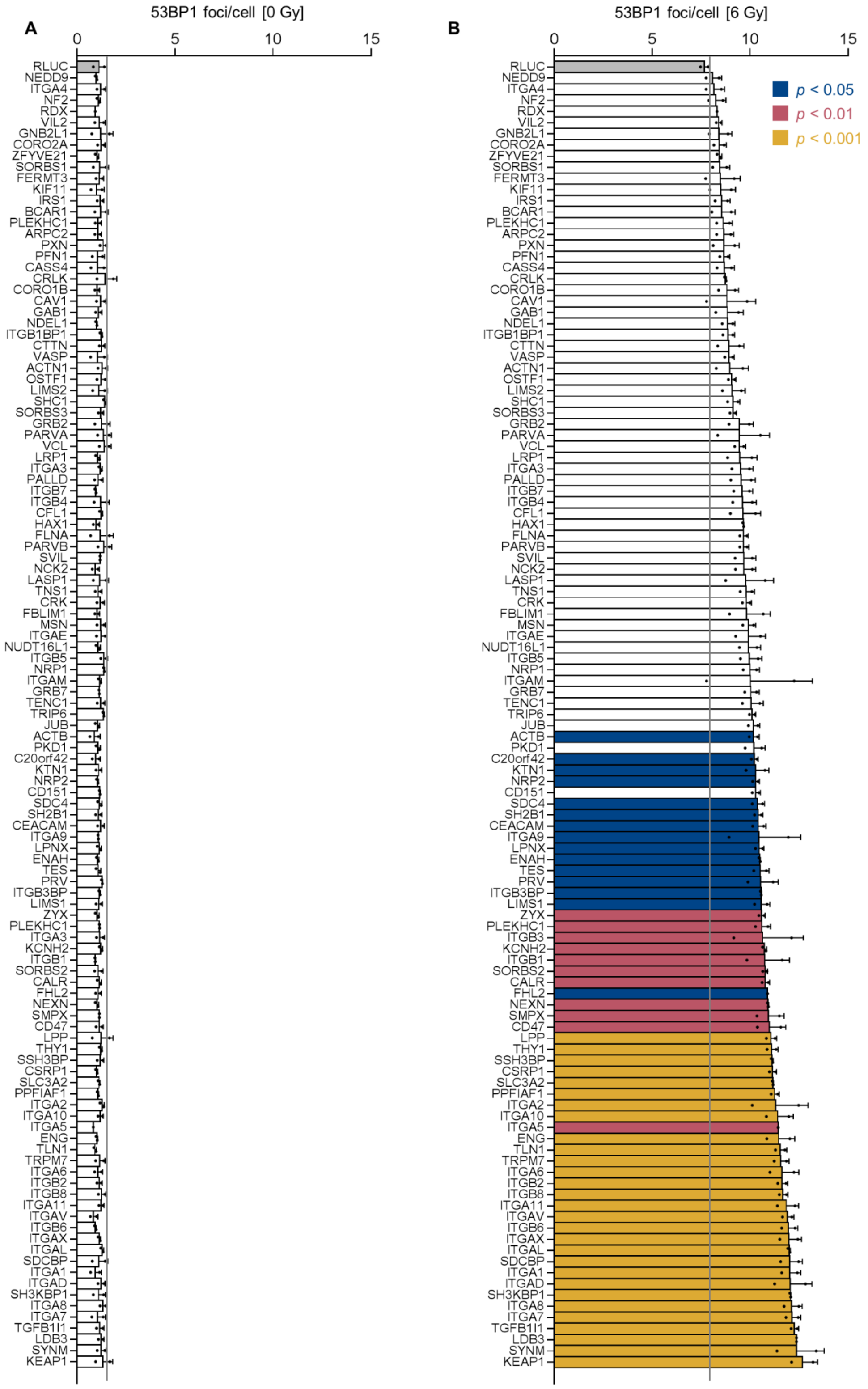

Figure S4. Identification of focal adhesion proteins (FAPs) affecting DNA repair. (**A**) Residual 53BP1 foci numbers (24 h after irradiation) in unirradiated cells upon indicated FAP knockdowns. (**B**) Residual 53BP1 foci numbers in 6-Gy irradiated cells upon indicated FAP knockdowns. Data are presented as mean ± SD (n = 4; two-sided t-test; * $p < 0.05$, ** $p < 0.01$, *** $p < 0.001$).

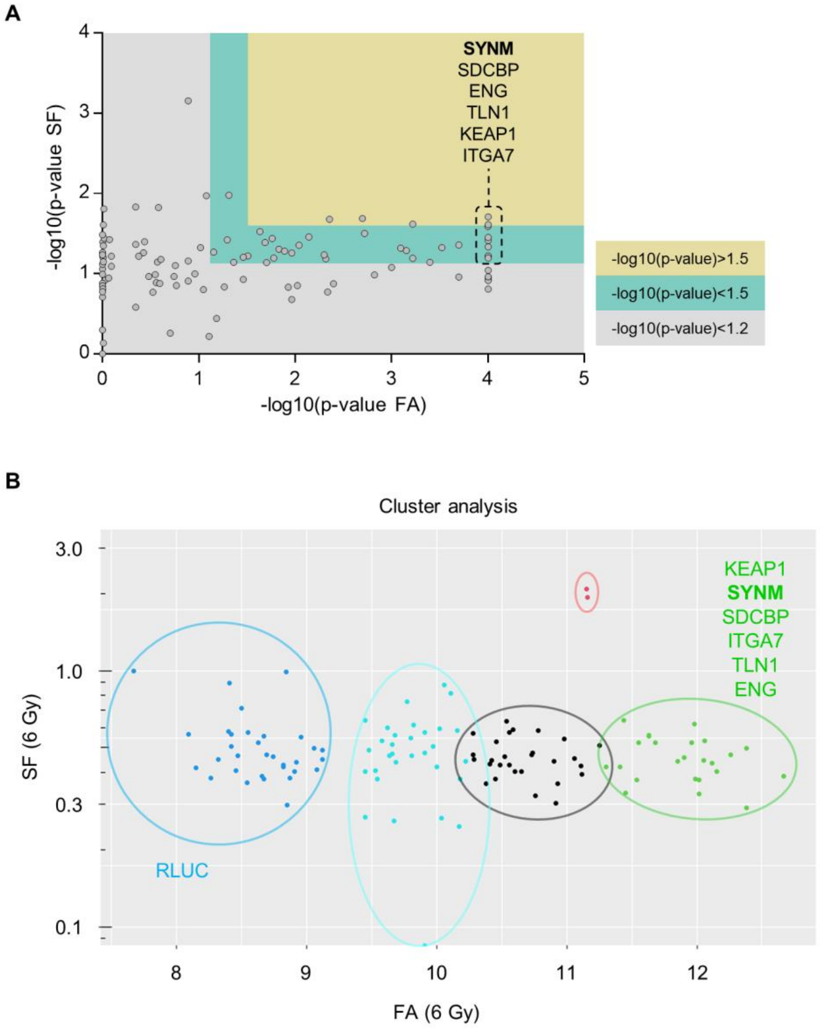

Figure S5. Identification of focal adhesion proteins affecting cell survival, radiosensitivity and DNA repair. (**A**) Scatter plot displaying the –log10(p-value) of 53BP1 residual foci/cell (FA, foci assay) and surviving fraction (SF) upon FAP knockdown and 6 Gy irradiation in UTSCC15 expressing EGFP-53BP1. Main selected candidates with high p-values are indicated. (**B**) Cluster analysis of screen results after x-ray exposure by K-means algorithm showed silenced genes are clustered in 5 groups with similar responses.

A

		DNA copy number alterations	
		Amplification	Deletion
	Total Dataset	+	-
HNSCC	HPV+	-	-
	HPV-	+	-
LUSC		+	-
CESC		+	-

B

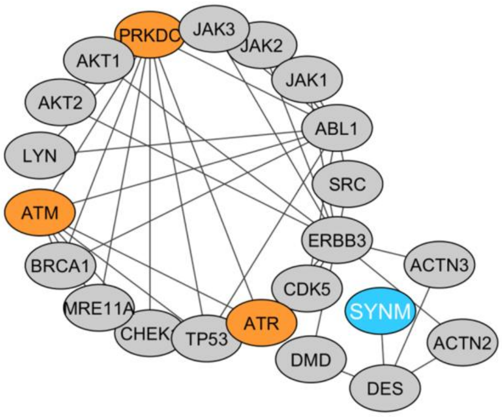

C

D

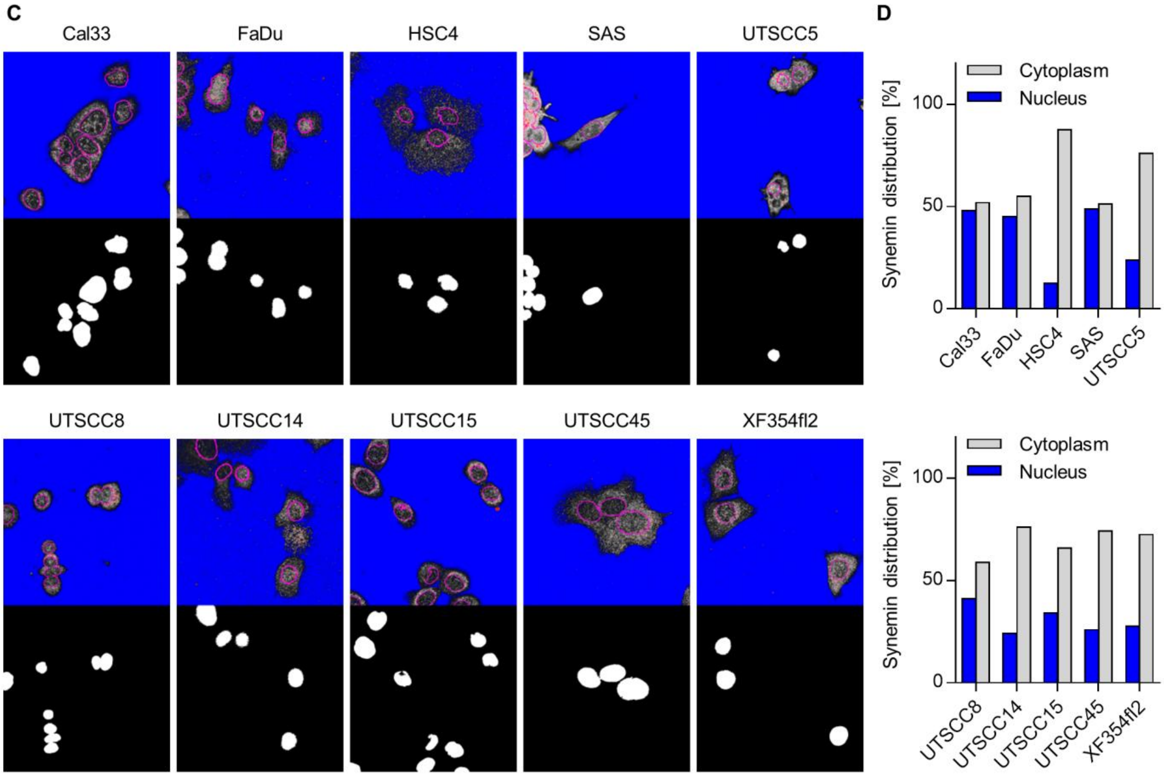

Figure S6. Altered expression of synemin expression in HNSCC. (**A**) DNA copy number alterations from TCGA data (Comprehensive genomic characterization of head and neck squamous cell carcinomas, HNSCC, The Cancer Genome Atlas Network; HPV, human papilloma virus), LUSC (lung squamous cell carcinoma), and CESC (cervical squamous cell carcinoma). (**B**) Interactome map of synemin using Cytoscape software (https://cytoscape.org/) with reactome plugin. (**C**) Fiji analysis of confocal images showing synemin distribution in all used HNSCC cell lines. (**D**) Graphs representing synemin distribution in the cell. Ratio of mean fluorescence intensity of nuclear to cytoplasmic localization was determined using the Intensity Ratio Nuclei Cytoplasm Tool plugin (NIH, USA).

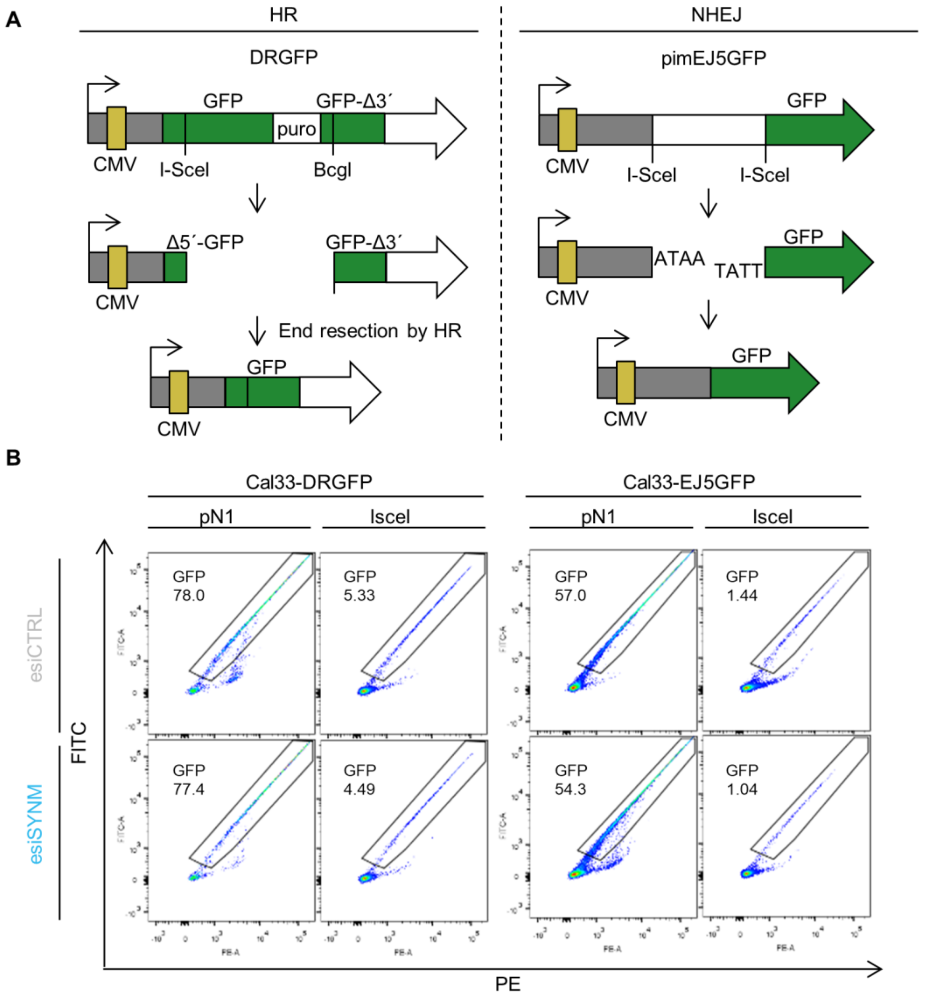

Figure S7. Synemin functions in NHEJ but not HR. (**A**) Schematic structure of the HR and NHEJ substrates DRGFP and pimEJ5GFP, respectively. The DRGFP presents two non-functional GFP copies and, when DBS are repaired, it results in a functional GFP. The pimEJ5GFP has an insertion between the CMV promoter and GFP preventing its translation. Once the endonuclease I-SceI cuts in the proper sites it generates a DSB that can be repair by NHEJ. (**B**) Representative dot plot figures of DNA repair reporter assay to evaluate homologous recombination (HR) and non-homologous end joining (NHEJ) activity in Cal33 transfectants. pN1 is the empty vector (pEGFP-N1) serving as positive control and I-SceI is a plasmid expressing an endonuclease used to generate the DSBs. Cells with proficient conduction of DNA repair express a GFP fluorescent protein.

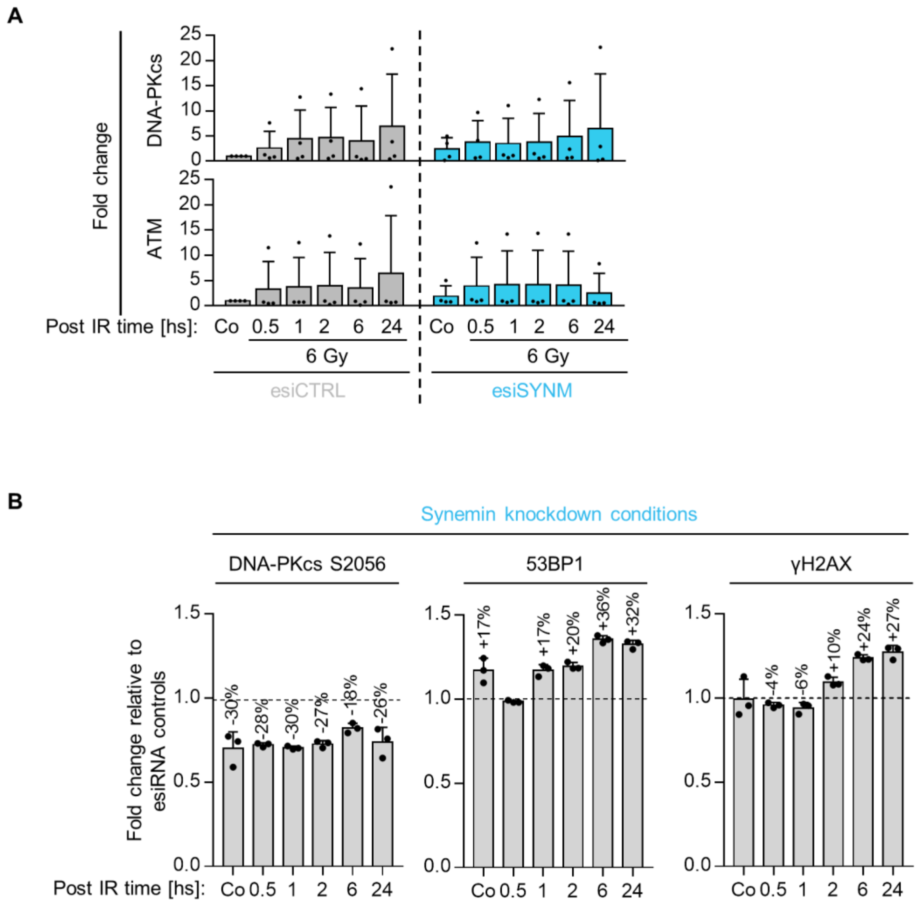

Figure S8. Synemin functions in non-homologous end joining. (**A**) Fold changes from synemin-depleted and 6-Gy irradiated SAS cells showing total forms of DNA-PKcs and ATM as detected by Western blotting on whole cell lysates. β-actin served as loading control (n = 4). (**B**) Normalized kinetics of DNA-PKcs S2056, 53BP1 and γH2AX foci insynemin knockdown cell cultures relative to controls at different time points post 1-Gy X-rays (n = 3).

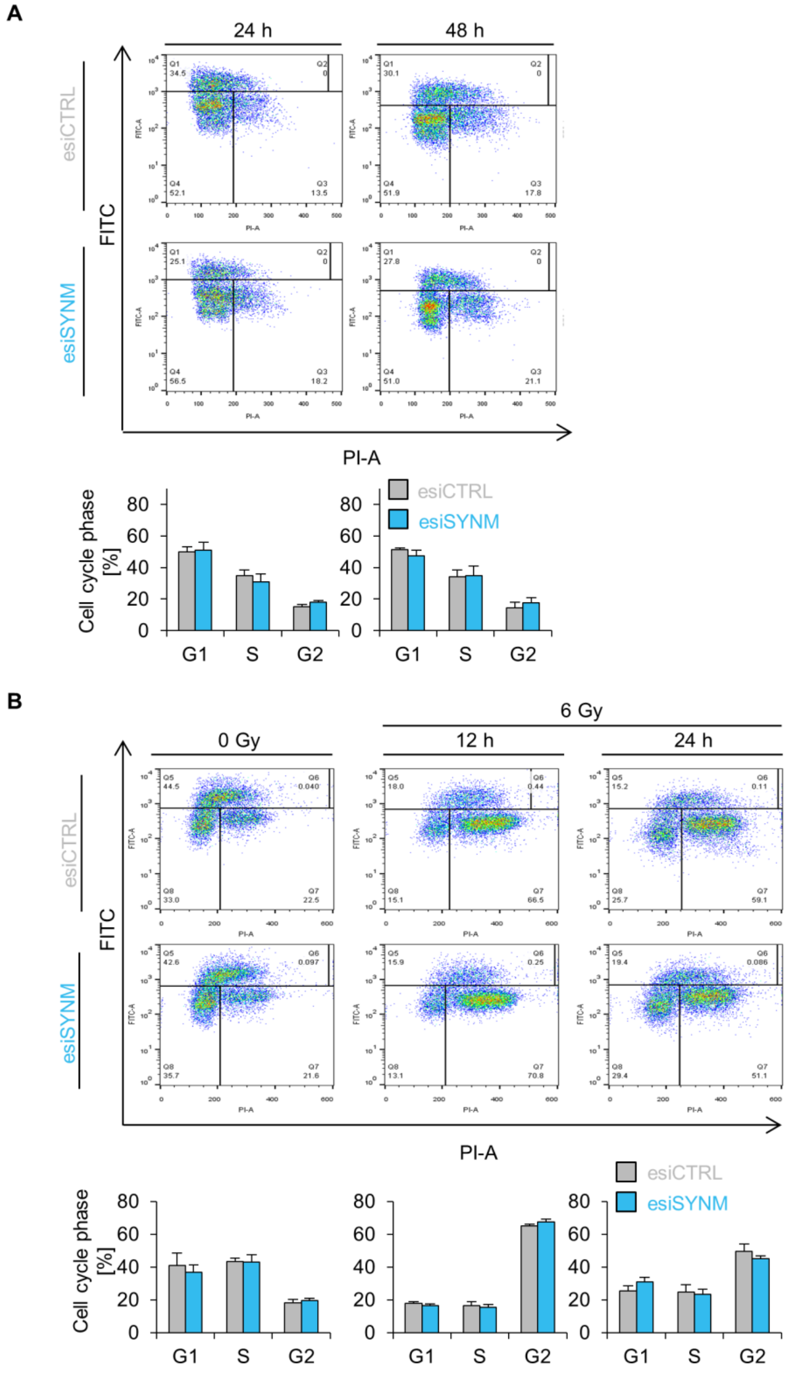

Figure S9. Function of synemin in cell cycling. (**A**) Cell cycle distribution of SAS cells upon synemin knockdown was measured by flow cytometry at 24 and 48 h post transfection (**B**) and at 12 and 24 h post 6 Gy X-ray irradiation. Data are presented as mean ± SD (n = 3; two-sided t-test).

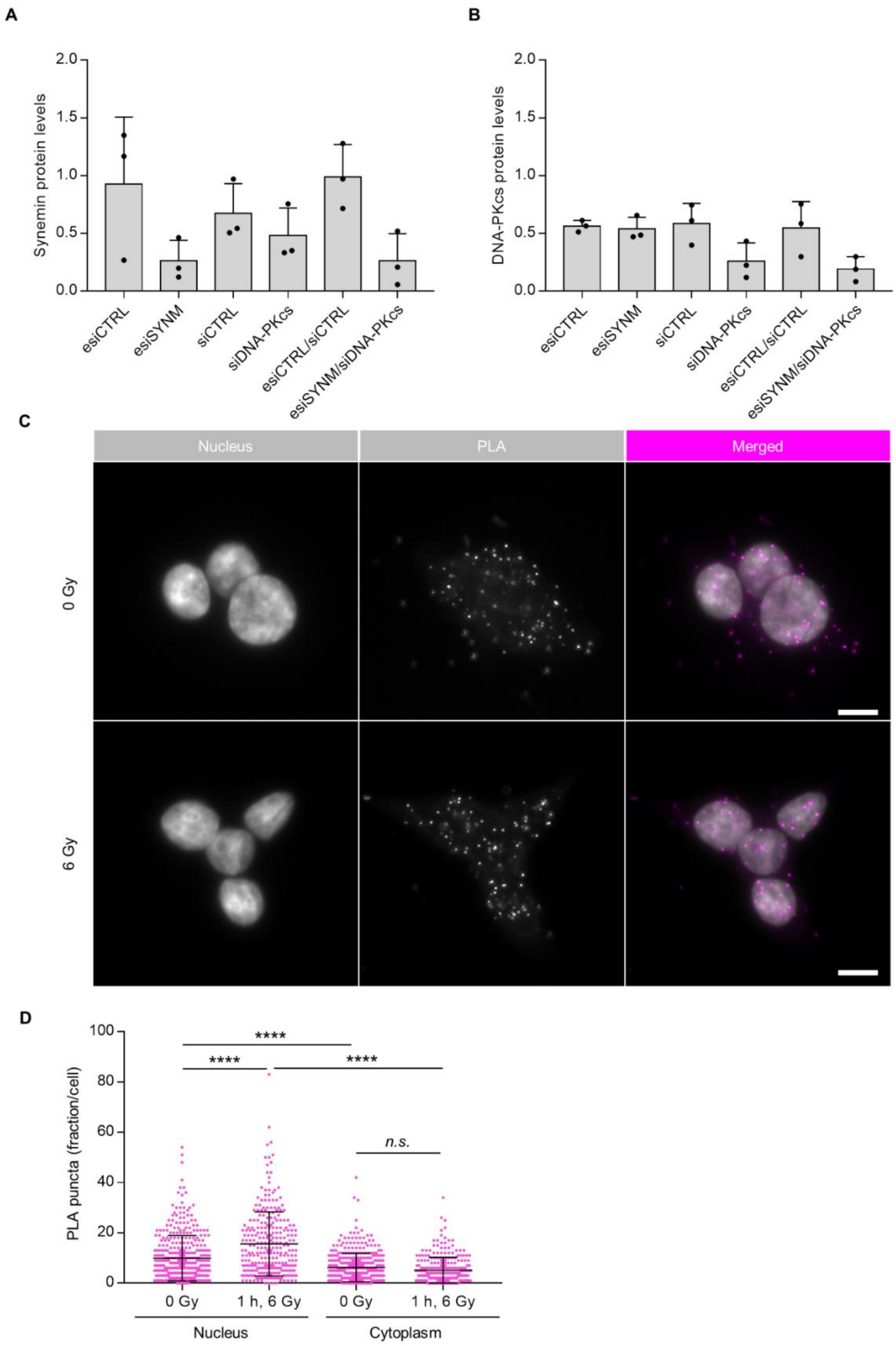

Figure S10. Synemin/DNA-PKcs co-control DSB repair and co-interact with each other. (**A, B**) Densitometries of knockdown efficiencies of single and double esi/siRNA transfections of SAS cells as detected by Western blotting on whole cell lysates. β-actin served as loading control (n = 3). (**C**) Interaction of synemin and DNA-Pkcs S2056 in unirradiated and irradiated SAS cells defined by proximity ligation assay (bar, 10 μm). (**D**) Quantification of PLA puncta in nucleus and cytoplasm using Fiji (n = 3; One-way ANOVA followed by post hoc test (Tukey multiple comparisons); ****$p < 0.0001$; n.s., not significant ($p \geq 0.05$)).

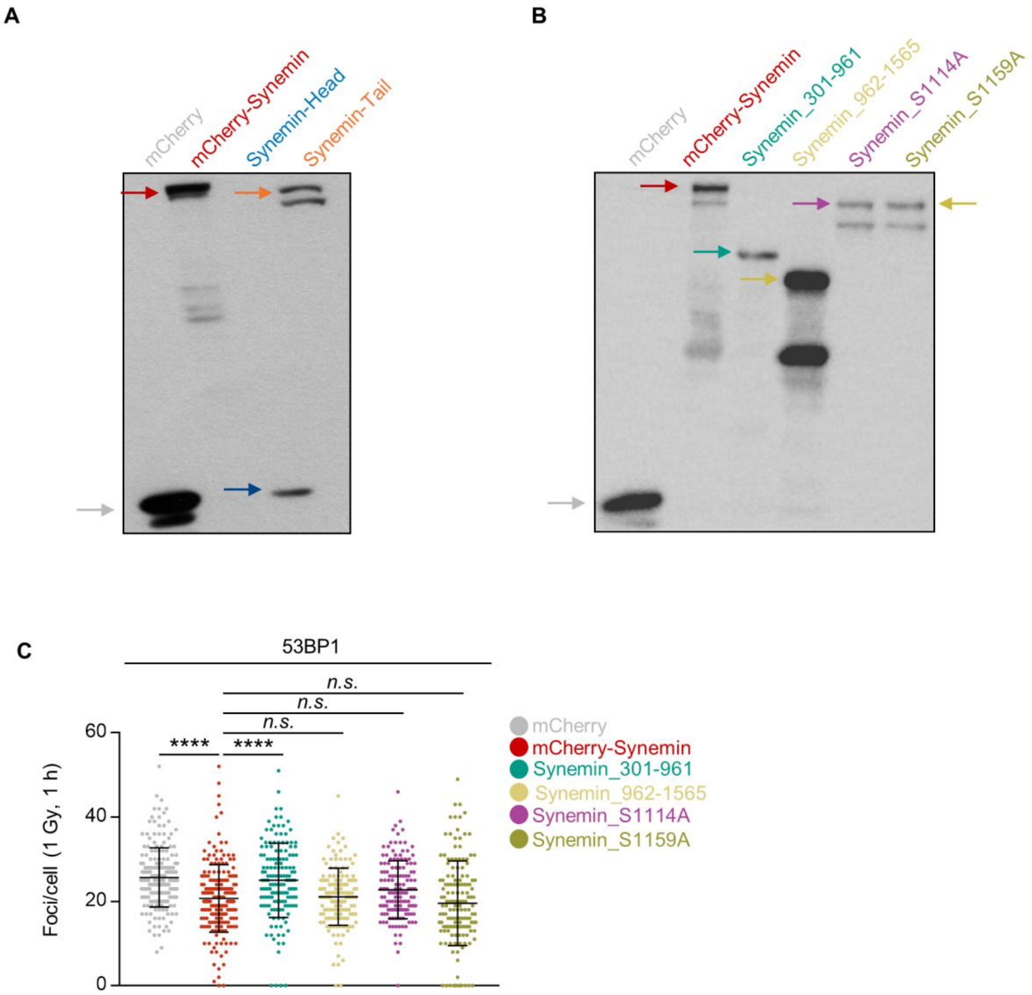

Figure S11. Expression of different Synemin constructs. (**A**) Western blotting of lysates from SAS cells expressing mCherry, mCherry-Synemin wildtype, mCherry-Synemin_Head, mCherry-Synemin_Tail. (**B**) Western blotting of lysates from SAS cells expressing mCherry, mCherry-Synemin wildtype, mCherry-Synemin_301-961, mCherry-Synemin_962-1565, mCherry-Synemin_S1114A and mCherry-Synemin_S1159A. Colored arrows indicate the synemin construct with the corresponding molecular weight. (**C**) 53BP1 foci after 1 h post 1-Gy X-ray exposure in SAS transfectants expressing mCherry-Synemin wildtype, mCherry-Synemin_301-961, mCherry-Synemin_962-1565, mCherry-Synemin_S1114A and mCherry-Synemin_S1159A (mCherry was used as control) (n = 3; One-way ANOVA followed by post hoc test (Tukey multiple comparisons); **** $p < 0.0001$; n.s., not significant ($p \geq 0.05$)).

Figure 2A

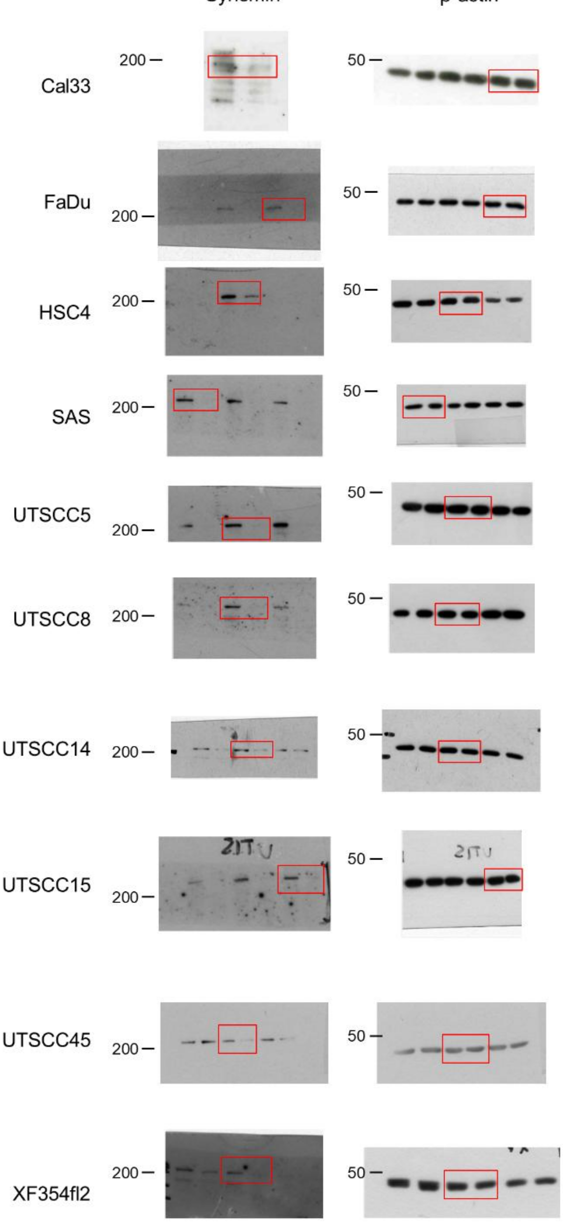

Figure 2J

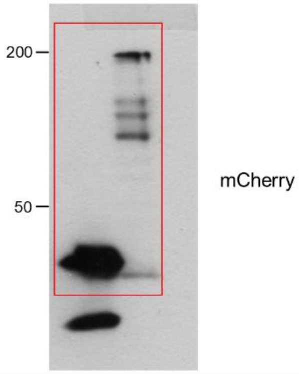

Figure 3D

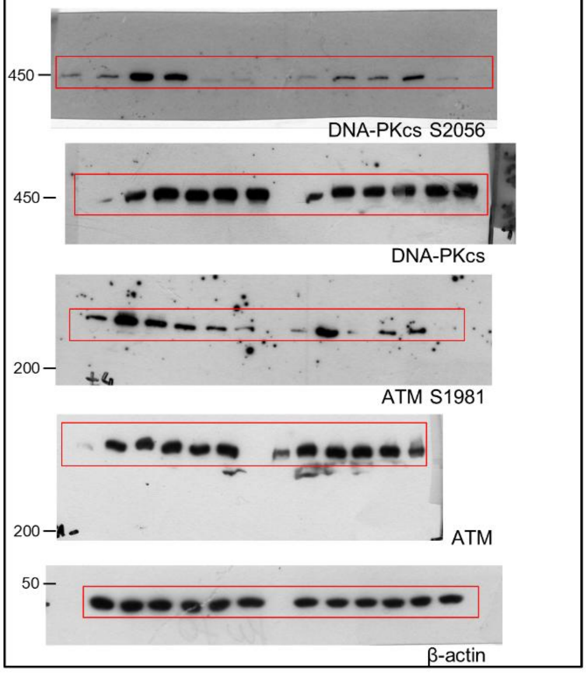

Figure 3F

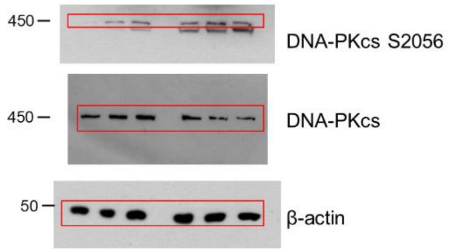

Figure 4A

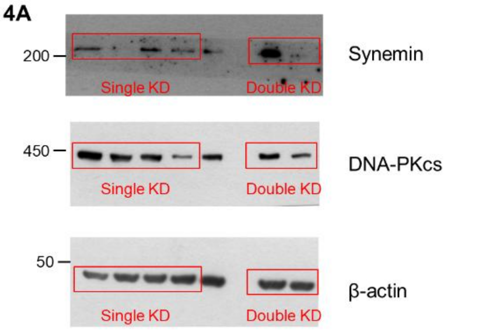

Figure 5A

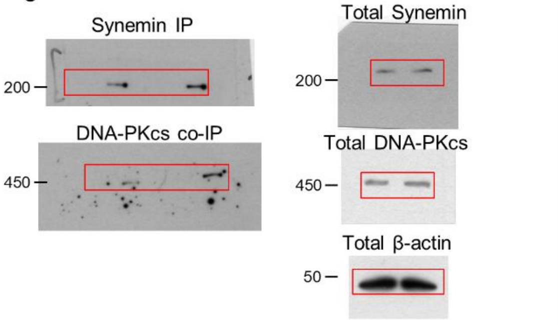

Figure 5C

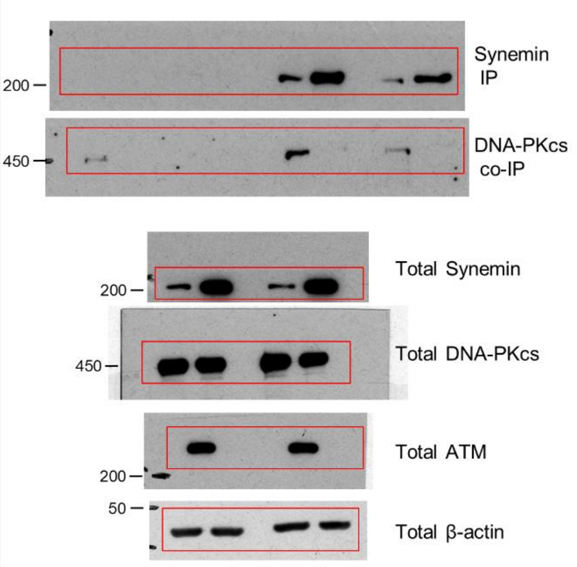

Figure 5B

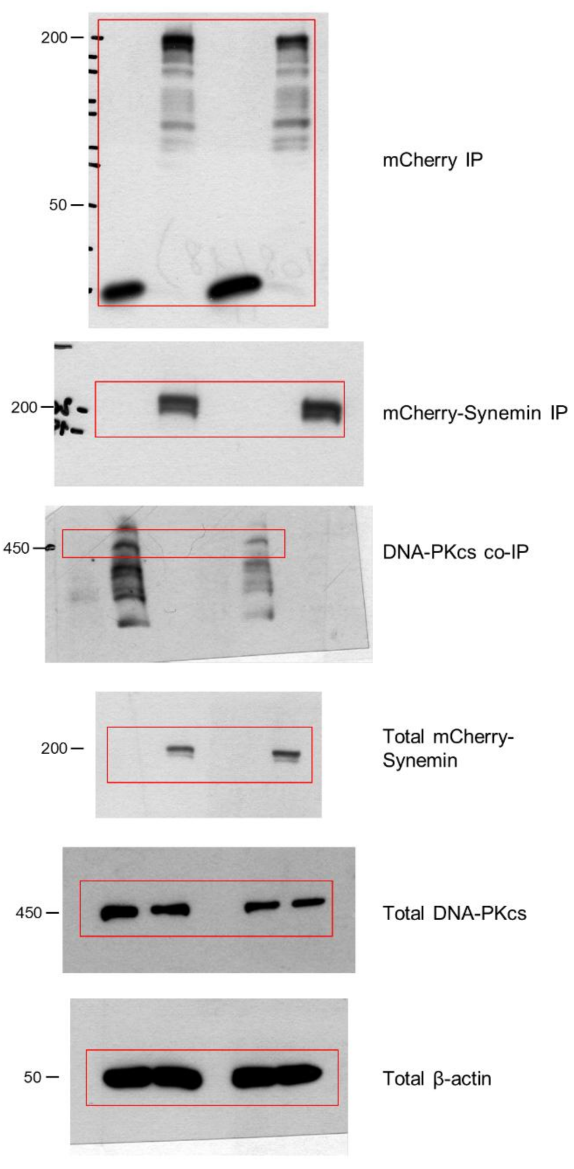

Figure 6F

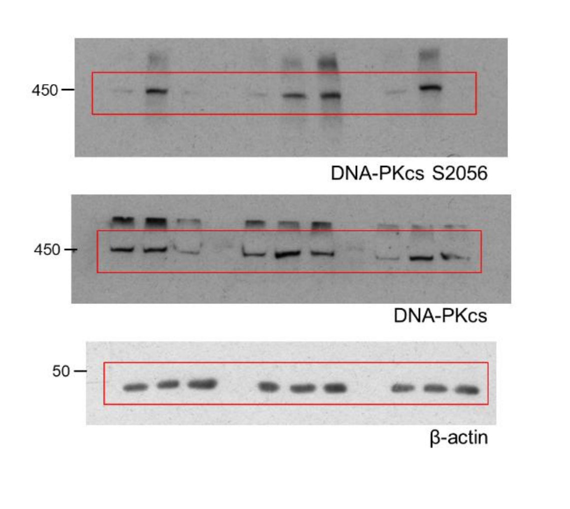

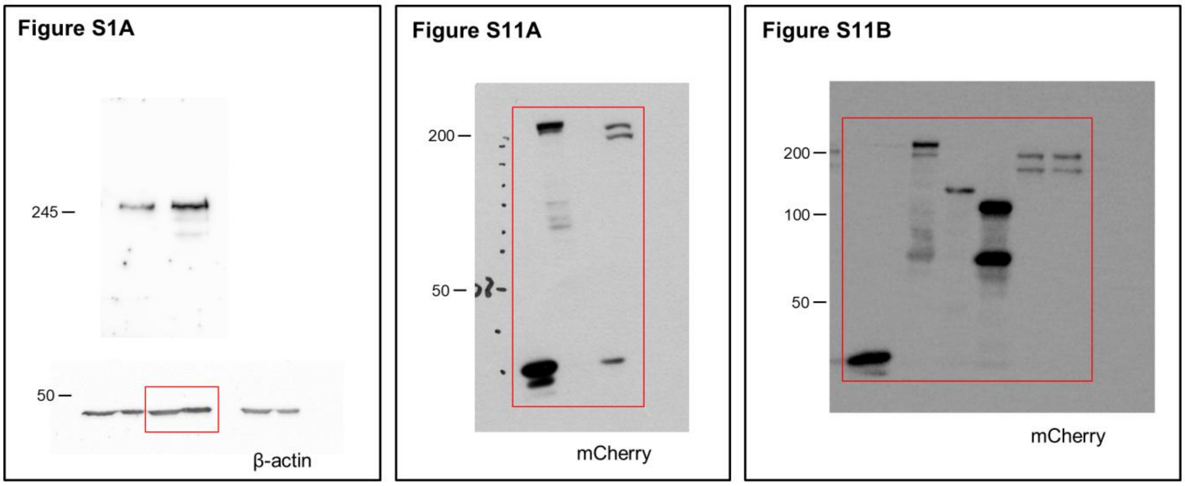

Figure S12. Compilation of uncropped immunoblots for all the figures. The corresponding main figures where the cropped versions are displayed are specified on top of the blots. The red rectangles highlight the areas of the plots that were used. The molecular weight standard [kDa] per blot is located on the left. The protein detected is written nearby the blots.

Supplementary Tables

Table S1. List of focal adhesion proteins selected for the screen library according to the Integrin Adhesome described by Horton et al., 2015.

ACTB	FBLIM1	ITGA9	KIF11	PARVA	SSH3BP
ACTN1	FERMT3	ITGAD	KTN1	PARVB	SVIL
ARPC2	FHL2	ITGAE	LASP1	PFN1	SYNM
BCAR1	FLNA	ITGAL	LDB3	PKD1	TENC1
C20orf42	GAB1	ITGAM	LIMS1	PLEKHC1	TES
CALR	GNB2L1	ITGAV	LIMS2	PPFIA1	TGFB1I1
CASS4	GRB2	ITGAX	LPP	PVR	THY1
CAV1	GRB7	ITGB1	LPXN	PXN	TLN1
CD151	HAX1	ITGB1BP1	LRP1	RDX	TNS1
CD47	IRS1	ITGB2	MSN	RLUC	TRIP6
CEACAM1	ITGA1	ITGB3	NCK2	SDC4	TRPM7
CFL1	ITGA10	ITGB3BP	NDEL1	SDCBP	VASP
CORO1B	ITGA11	ITGB4	NEDD9	SH2B1	VCL
CORO2A	ITGA2	ITGB5	NEXN	SH3KBP1	VIL2
CRK	ITGA3	ITGB6	NF2	SHC1	ZFYVE21
CRKL	ITGA4	ITGB7	NRP1	SLC3A2	ZYX
CSRP1	ITGA5	ITGB8	NRP2	SMPX	
CTTN	ITGA6	JUB	NUDT16L1	SORBS1	
ENAH	ITGA7	KCNH2	OSTF1	SORBS2	
ENG	ITGA8	KEAP1	PALLD	SORBS3	

Table S2. Enhancement ratios of the plating efficiency of cells under FAP library knockdown. The table contains the mean and the p-value for each silenced protein.

Enhancement Ratio (0 Gy)								
**FAP**	**Mean**	***p*-value**	**FAP**	**Mean**	***p*-value**	**FAP**	**Mean**	***p*-value**
**ACTB**	0.218787	0.296738	**ITGAL**	0.620638	0.344969	**PPFIA1**	0.485256	0.077743
**ACTN1**	0.890437	0.663467	**ITGAM**	0.666169	0.474556	**PVR**	0.842457	0.381801
**ARPC2**	0.723471	0.505013	**ITGAV**	1.287555	0.242222	**PXN**	0.450333	0.089756
**BCAR1**	1.092557	0.826502	**ITGAX**	0.451351	0.086505	**RDX**	0.379812	0.121085
**C20orf42**	1.00201	0.994049	**ITGB1**	0.535977	0.203078	**RLUC**	1	1
**CALR**	0.760815	0.402756	**ITGB1BP1**	1.378742	0.407063	**SDC4**	1.630541	0.191021
**CASS4**	0.540939	0.342177	**ITGB2**	0.61205	0.333057	**SDCBP**	0.542153	0.368684
**CAV1**	0.771447	0.30279	**ITGB3**	0.370594	0.115324	**SH2B1**	0.963286	0.909822
**CD151**	0.532127	0.250045	**ITGB3BP**	0.419493	0.045849	**SH3KBP1**	0.71892	0.42389
**CD47**	0.527273	0.108184	**ITGB4**	0.684614	0.164357	**SHC1**	0.870773	0.750854
**CEACAM1**	0.614071	0.386168	**ITGB5**	0.399693	0.201343	**SLC3A2**	0.744613	0.646514
**CFL1**	0.417186	0.063205	**ITGB6**	0.524212	0.166341	**SMPX**	0.407986	0.245538
**CORO1B**	0.762593	0.593817	**ITGB7**	0.623462	0.307414	**SORBS1**	0.562458	0.390378
**CORO2A**	0.431972	0.394586	**ITGB8**	0.709589	0.380879	**SORBS2**	0.807714	0.436095
**CRK**	0.732059	0.49421	**JUB**	1.246845	0.448609	**SORBS3**	0.633079	0.45497
**CRKL**	0.427692	0.131564	**KCNH2**	0.680588	0.333257	**SSH3BP**	0.551018	0.410779
**CSRP1**	0.156499	0.127136	**KEAP1**	0.774654	0.496029	**SVIL**	0.481987	0.208234
**CTTN**	0.58211	0.420259	**KIF11**	1.895178	0.026392	**SYNM**	0.282682	0.137298
**ENAH**	0.591132	0.251234	**KTN1**	0.510572	0.144869	**TENC1**	0.819651	0.647319
**ENG**	0.592445	0.505988	**LASP1**	0.56644	0.359162	**TES**	0.950824	0.375272
**FBLIM1**	1.189644	0.610917	**LDB3**	1.047985	0.759712	**TGFB1I1**	1.000344	0.998213
**FERMT3**	0.908059	0.750284	**LIMS1**	0.725507	0.256389	**THY1**	0.518769	0.090723
**FHL2**	0.740103	0.035586	**LIMS2**	0.619887	0.194736	**TLN1**	0.96443	0.891472
**FLNA**	0.787682	0.532634	**LPP**	0.722871	0.432133	**TNS1**	0.531372	0.272214
**GAB1**	0.728582	0.236164	**LPXN**	0.508352	0.122459	**TRIP6**	0.567396	0.024664
**GNB2L1**	1.684106	0.216224	**LRP1**	0.451535	0.15179	**TRPM7**	0.866785	0.726025
**GRB2**	1.023914	0.94941	**MSN**	2.135581	0.28631	**VASP**	0.90681	0.785223
**GRB7**	0.846479	0.629825	**NCK2**	0.932307	0.852757	**VCL**	0.811571	0.491124
**HAX1**	0.563375	0.233459	**NDEL1**	0.697475	0.354876	**VIL2**	0.700898	0.460385
**IRS1**	0.531866	0.320151	**NEDD9**	0.461269	0.208131	**ZFYVE21**	0.674791	0.15892
**ITGA1**	0.710938	0.332908	**NEXN**	0.325041	0.195018	**ZYX**	0.391948	0.002819
**ITGA10**	0.770097	0.446204	**NF2**	0.694538	0.494403			
**ITGA11**	0.844503	0.437838	**NRP1**	0.603221	0.267625			
**ITGA2**	0.595777	0.229198	**NRP2**	0.622675	0.278972			
**ITGA3**	1.021058	0.912004	**NUDT16L1**	0.538864	0.157145			
**ITGA4**	0.449399	0.310329	**OSTF1**	0.402063	0.011891			
**ITGA5**	0.294663	0.310628	**PALLD**	0.690447	0.232747			
**ITGA6**	0.663847	0.385743	**PARVA**	0.774387	0.562635			
**ITGA7**	0.630924	0.286633	**PARVB**	0.858168	0.610493			
**ITGA8**	0.516808	0.217457	**PFN1**	0.785057	0.584705			
**ITGA9**	0.637579	0.328443	**PKD1**	0.937738	0.747646			
**ITGAD**	0.497173	0.397707	**PLEKHC1**	0.486399	0.002196			
**ITGAE**	0.750931	0.268299	**PLEKHC1**	0.605436	0.268174			

Table S3. Enhancement ratios of surviving fraction of cells upon FAP knockdown and 6 Gy X-Ray exposure. The table contains the mean and the p-value for each silenced protein.

Enhancement Ratio (6 Gy)								
**FAP**	**Mean**	***p*-value**	**FAP**	**Mean**	***p*-value**	**FAP**	**Mean**	***p*-value**
**ACTB**	4.044148	0.00285	**ITGAL**	2.642642	0.049733	**PPFIA1**	1.956009	0.016185
**ACTN1**	1.81123	0.077887	**ITGAM**	1.687302	0.167701	**PVR**	1.575727	0.086803
**ARPC2**	2.573264	0.017091	**ITGAV**	2.182184	0.012616	**PXN**	2.639696	0.046944
**BCAR1**	1.737145	0.037327	**ITGAX**	1.616834	0.089632	**RDX**	2.22631	0.086487
**C20orf42**	2.263046	0.06362	**ITGB1**	2.09506	0.018522	**RLUC**	1	1
**CALR**	1.721035	0.087917	**ITGB1BP1**	2.619569	0.005127	**SDC4**	2.760266	0.020976
**CASS4**	2.137935	0.018984	**ITGB2**	1.795348	0.06032	**SDCBP**	2.665955	0.009358
**CAV1**	2.380232	0.0129	**ITGB3**	2.483497	0.010183	**SH2B1**	2.324003	0.010252
**CD151**	2.217147	0.029769	**ITGB3BP**	2.344994	0.022192	**SH3KBP1**	1.875269	0.038054
**CD47**	1.842779	0.033364	**ITGB4**	1.66912	0.08802	**SHC1**	2.051661	0.021981
**CEACAM1**	2.23503	0.047882	**ITGB5**	1.971374	0.060857	**SLC3A2**	0.517665	0.62181
**CFL1**	2.147715	0.189955	**ITGB6**	1.915353	0.085165	**SMPX**	2.758955	0.014706
**CORO1B**	2.334509	0.028761	**ITGB7**	1.872167	0.044907	**SORBS1**	2.447648	0.042133
**CORO2A**	1.975312	0.043732	**ITGB8**	1.912818	0.03554	**SORBS2**	3.074549	0.009055
**CRK**	2.145052	0.039019	**JUB**	1.709387	0.038637	**SORBS3**	2.215941	0.02644
**CRKL**	2.166548	0.028248	**KCNH2**	2.122255	0.014384	**SSH3BP**	2.546005	0.037913
**CSRP1**	0.479178	0.564995	**KEAP1**	2.582338	0.012831	**SVIL**	1.787962	0.1016
**CTTN**	2.472528	0.006822	**KIF11**	1.402887	0.14792	**SYNM**	3.433697	0.015989
**ENAH**	2.341327	0.017542	**KTN1**	2.136976	0.018346	**TENC1**	1.137676	0.623733
**ENG**	2.992243	0.010865	**LASP1**	1.318115	0.597581	**TES**	2.171149	0.012378
**FBLIM1**	1.633571	0.184407	**LDB3**	2.003155	0.138989	**TGFB1I1**	2.132824	0.017184
**FERMT3**	2.152705	0.014594	**LIMS1**	1.738436	0.309606	**THY1**	2.364349	0.012103
**FHL2**	2.258673	0.024298	**LIMS2**	2.43107	0.009139	**TLN1**	2.663803	0.010139
**FLNA**	2.115305	0.026057	**LPP**	2.202217	0.028634	**TNS1**	1.82747	0.030197
**GAB1**	1.008874	0.875554	**LPXN**	1.892749	0.035443	**TRIP6**	1.226288	0.408417
**GNB2L1**	1.122442	0.376171	**LRP1**	2.043618	0.042298	**TRPM7**	1.917565	0.039823
**GRB2**	2.48485	0.026327	**MSN**	0	0.00048	**VASP**	2.270053	0.045936
**GRB7**	3.769021	0.002237	**NCK2**	2.280599	0.073647	**VCL**	3.743628	0.007878
**HAX1**	1.949069	0.147178	**NDEL1**	3.358212	0.004183	**VIL2**	1.731482	0.099372
**IRS1**	2.741913	0.009604	**NEDD9**	1.777788	0.09752	**ZFYVE21**	1.776298	0.19275
**ITGA1**	3.030718	0.008654	**NEXN**	3.289021	0.008161	**ZYX**	1.695661	0.083168
**ITGA10**	2.378205	0.008409	**NF2**	2.628556	0.105819			
**ITGA11**	2.25142	0.029782	**NRP1**	2.367223	0.015286			
**ITGA2**	2.374183	0.012622	**NRP2**	1.763388	0.034822			
**ITGA3**	2.454615	0.051182	**NUDT16L1**	1.738727	0.144331			
**ITGA4**	2.388571	0.048194	**OSTF1**	2.016447	0.182003			
**ITGA5**	1.566164	0.060344	**PALLD**	2.645041	0.023753			
**ITGA6**	1.784018	0.042916	**PARVA**	1.562069	0.318587			
**ITGA7**	2.482874	0.020138	**PARVB**	3.86016	0.011911			
**ITGA8**	2.301613	0.031814	**PFN1**	1.803768	0.150091			
**ITGA9**	2.641605	0.022245	**PKD1**	2.650614	0.02794			
**ITGAD**	2.241082	0.037898	**PLEKHC1**	1.911817	0.033548			
**ITGAE**	2.090224	0.032749	**PLEKHC1**	2.476154	0.023976			

Table S4. Primary antibodies.

Antibody	Application	Dilution	Company
ATM, rabbit, monoclonal	Western blot<br>Immunoprecipitation	1:1000<br>10 μl	Abcam, Cambridge, UK
ATM S1981, mouse, monoclonal	Western blot	1:500	Rockland, Pennsylvania, USA
β-Actin, Klon AC-15, mouse, monoclonal	Western blot	1:10000	Sigma Aldrich, Taufkirchen, Germany
DNA-PKcs, rabbit, polyclonal	Western blot<br>Immunoprecipitation	1:1000<br>10 μl	Cell Signaling, Frankfurt a. M., Germany
DNA-PKcs S2056, rabbit, polyclonal	Western blot<br>Immunofluorescence<br>PLA	1:500<br>1:200<br>1:100	Abcam, Cambridge, UK
Ku70, mouse, monoclonal	Western blot	1:1000	Abcam, Cambridge, UK
γH2AX S139, mouse, monoclonal	Western blot<br>Immunofluorescence	1:1000<br>1:200	Millipore, Massachusetts, USA
53BP1, rabbit, polyclonal	Immunofluorescence	1:200	Novus Biologicals, Colorado, USA
Desmuslin, mouse, monoclonal	Western blot<br>PLA	1:500<br>1:100	Abcam, Cambridge, UK
Desmuslin, rabbit, polyclonal	Western blot	1:500	Abcam, Cambridge, UK
Desmuslin, mouse, monoclonal	Immunoprecipitation<br>Immunofluorescence	3.5 μg<br>1:100	Santa Cruz, Dallas, USA
BrdU, mouse, monoclonal	FACs Analysis	1:50	BD, Heidelberg, Germany
PARP1, rabbit, polyclonal	Western blot	1:1000	Cell Signaling, Frankfurt a. M., Germany
Synemin	Western blot zebrafish	1:1000	Sigma-Aldrich, Taufkirchen, Germany
mCherry	Western blot<br>Immunoprecipitation	1:1000<br>5 μl	Abcam, Cambridge, UK
Phosphoserine	Western blot	1:250	Abcam, Cambridge, UK

Table S5. Secondary antibodies.

Antibody	Application	Dilution	Company
Anti-mouse IgG, HRP conjugated	Western blot	1:5000	Pierce, Bonn, Germany
Anti-rabbit IgG, HRP conjugated	Western blot	1:5000	Pierce, Bonn, Germany
Anti-mouse IgG, HRP conjugated	Immunoprecipitation	1:1000	GeneTex, Irvine, USA
Anti-rabbit IgG, HRP conjugated	Immunoprecipitation	1:1000	GeneTex, Irvine, USA
Alexa Fluor®488 Anti-mouse IgG	Immunofluorescence	1:200	Life Technologies GmbH, Darmstadt, Germany
Alexa Fluor®488 Anti-rabbit IgG	Immunofluorescence	1:200	Life Technologies GmbH, Darmstadt, Germany
Alexa Fluor®594 Anti-mouse IgG	Immunofluorescence	1:200	Life Technologies GmbH, Darmstadt, Germany
Alexa Fluor®594 Anti-rabbit IgG	Immunofluorescence	1:200	Life Technologies GmbH, Darmstadt, Germany
Alexa Flour®594 Phalloidin	Immunofluorescence	1:800	Life Technologies GmbH, Darmstadt, Germany

Table S6. RNAi sequences and morpholinos.

RNAi	Sequence (Sense)	Company
esiRLUC	5′-ATTTATTAATTATTATGATCAGAAAAACATGCAGAAAATGCTGTTATTTTTTAC-3′	Eupheria Biotech, Dresden, Germany
esiSYNM	5′-AAACAGACCAGAAACCATCCGAACAAAGCCAGAA-GAGAAAATGTTCGATTCTAA-3′	Eupheria Biotech, Dresden, Germany
siCTRL#1	5′- AAAACAGUUGCGCAGCCUGAAtt-3′	MWG Eurofins, Ebersberg, Germany
siDNA-PKcs	5′-GGCAAUUCGUCCUCAGAUUtt-3′	MWG Eurofins, Ebersberg, Germany

Table S7. Primers.

Primer	Sequence (Sense)	Company
Synemin ΔLinker-Tail-F	5′-AGCTTcgATGCTGTCCTGGCGGCTGCAGACGGGCCCCG-3′	MWG Eurofins, Ebersberg, Germany
Synemin ΔLinker-Tail-R	5′-AgcTACGACAGGACCGCCGACGTCTGCCCGGGGCCTAG-3′	MWG Eurofins, Ebersberg, Germany
Synemin ΔHead-Linker-F	5′-cccAAGCTTcg-GTGAAGACCGGCCTCAGTCTGG-3′	MWG Eurofins, Ebersberg, Germany
Synemin ΔHead-Linker-R	5′-cgcGGATCC-TTAAAACCAATGCCCATCATTCTC-3′	MWG Eurofins, Ebersberg, Germany
Synemin_S1114A-F	CCACAGGCTTTGCCCAGTCACAGGTGCTGGAGGATG-F	MWG Eurofins, Ebersberg, Germany
Synemin_S1114A-R	CCACAGGCTTTGCCCAGGCACAGGTGCTGGAGGATG-R	MWG Eurofins, Ebersberg, Germany
Synemin_S1159A-F	GCGGGAGGTGACCTAGCTCAGGCAGCGAGCCCGACC-F	MWG Eurofins, Ebersberg, Germany
Synemin_S1159A-R	GGTCGGGCTCGCTGCCTGAGCTAGGTCACCTCCCGC-R	MWG Eurofins, Ebersberg, Germany

Supplementary References

1. Dickreuter, E.; Eke, I.; Krause, M.; Borgmann, K.; van Vugt, M.A.; Cordes, N. Targeting of β1 integrins impairs DNA repair for radiosensitization of head and neck cancer cells. *Oncogene* **2016**, *35*, 1353–1362. doi:10.1038/onc.2015.212.
2. Eke, I.; Deuse, Y.; Hehlgans, S.; Gurtner, K.; Krause, M.; Baumann, M.; Shevchenko, A.; Sandfort, V.; Cordes, N. β 1 Integrin/FAK/cortactin signaling is essential for human head and neck cancer resistance to radiotherapy. *J. Clin. Invest.* **2012**, *122*, 1529–1540. doi:10.1172/JCI61350.
3. Horton, E.R.; Byron, A.; Askari, J.a.; Ng, D.H.J.; Millon-Frémillon, A.; Robertson, J.; Koper, E.J.; Paul, N.R.; Warwood, S.; Knight, D.; et al. Definition of a consensus integrin adhesome and its dynamics during adhesion complex assembly and disassembly. *Nat. Cell Biol.* **2015**, *17*, 1577–1587, doi:10.1038/ncb3257.
